# Retraction: The extract from the roots of *Rose odorata sweet* var. *gigantean* (Coll. et Hemsl.) Rehd. et Wils attenuates DSS-induced ulcerative colitis by regulating the Nrf2/NF-κB signaling pathways

**DOI:** 10.1039/d4ra90120g

**Published:** 2024-10-07

**Authors:** Xinyu Li, Rui Liu, Yanmin Zhao, Naying Gao, Xin Jin, Xiaoxia Gao, Tan Li, Dailin Liu

**Affiliations:** a Tianjin University of Traditional Chinese Medicine Tianjin 300193 China; b Tianjin Key Laboratory for Prevention and Control of Occupational and Environmental Hazard, Logistics University of Chinese People’s Armed Police Force Tianjin 300309 P. R. China dailinlch@163.com Tanli20042001@163.com +86-22-84876608 +86-22-84876589 +86-22-84876608 +86-22-84876589; c Department of Traditional Chinese Medicine, Guangdong Pharmaceutical College Guangdong 510006 P. R. China

## Abstract

Retraction of ‘The extract from the roots of *Rose odorata sweet* var. *gigantean* (Coll. et Hemsl.) Rehd. et Wils attenuates DSS-induced ulcerative colitis by regulating the Nrf2/NF-κB signaling pathways’ by Xinyu Li *et al.*, *RSC Adv.*, 2020, **10**, 9450–9461, https://doi.org/10.1039/C9RA10747A.

The Royal Society of Chemistry hereby wholly retracts this *RSC Advances* article due to concerns with the image within Fig. 5A, the reliability of the western blot data, and unattributed text overlap with ref. 1.

In Fig. 5A, the ROE (250 mg kg^−1^) column on the top (NrF2) line and bottom (NF-KB) line appear to have similarities consistent with the image being flipped and rotated.

The raw data for the western blot panels in Fig. 7 have no visible background and appear over-contrasted. The individual bands are uncharacteristically very uniform and regular in shape, indicating that they may not be genuine.

Given the significance of the concerns about the validity of both the data in the article and the raw data provided by the authors, the findings presented in this paper are not reliable.

The authors were informed about the retraction of this article but have not responded.

Laura Fisher, Executive Editor, *RSC Advances*

22nd August 2024
